# Computer aided pharmacokinetic profiling and toxicity analysis of naphthalene

**DOI:** 10.6026/97320630017080

**Published:** 2021-01-31

**Authors:** KSV Angu Bala Ganesh, Kesavi Durairaj, Gopinathan Narasimhan, Kalaivani Periyathambi

**Affiliations:** 1 Research Fellow, Department of Anatomy, Sri Ramachandra Medical College and Research Institute, SRIHER-DU, Porur, Chennai-116,Tamil Nadu, India; 2 Professor, Department of Anatomy, Sri Ramachandra Medical College and Research Institute, SRIHER-DU, Porur,Chennai-116, Tamil Nadu, India; 3 Assistant Professor, Department of Pharmaceutical Chemistry, Faculty of Pharmacy, SRIHER-DU, Porur,Chennai-116, Tamil Nadu, India; 4 Research Scientist, Centre for Toxicology and Developmental Research (CEFTE), SRIHER-DU, Porur,Chennai-116, Tamil Nadu, India;

**Keywords:** Naphthalene, neurotoxicity, prediction data

## Abstract

Naphthalene is an aromatic hydrocarbon used as room freshner. Therefore, it is of interest to document the computer aided pharmacokinetic profiling and toxicity analysis data of naphthalene.

## Background

Naphthalene (PubChem Id-931) is a polynuclear arenes having a pungent smell with a density of ≈0.44 mg/m3 in air [1]. The sources in the environment are abundant of this aromatic hydrocarbon. Domestic exposure of this naphthalene is due to its use in form of mothballs [2]. Naphthalene exposure is also seen with cement-based materials, which can be a potential environmental exposure apart from mothballs [3]. There is an often misuse of naphthalene in contravention of the insect repellents mark. The National Pesticide Information Center found considerable incidents of mothball exposure during 2006, and majority incidents were due to misuse [4]. Frequent misuse includes air ducts or crawlspaces, where vapor can be reached all over the enclosed milieu. The unintentional pediatric contact to mothball is a concern [5]. Newborns were affected with hemolytic anemia due to naphthalene exposure [6]. Few studies demonstrated the toxicity of naphthalene exposure [7,8], but still there is a gap in profiling the compound. Therefore, it is of interest to document the computer aided pharmacokinetic profiling and toxicity analysis data of naphthalene.

## Materials and Methods:

QikProp tool is a fast, precise, easy-to-use in identification of drug like compounds through absorption, distribution, metabolism, and excretion (ADME) calculation software [9]. We used QikProp to calculate hydrogen bond donor and acceptor, molecular weight, octanol-water partition coefficient, skin permeability, MDCK cell permeability, humeral absorption and their position followed Lipinski's rule of five (ro5) [10]. The reference compounds with PubChem ID 15608 (Methyl Tridecanoate), 8181 (Methyl Palmitate), 5362717 (Methyl Petroselinate) and 931 is naphthalene is used.

## Results and Discussion:

The physical descriptors described in Table 1 (see PDF), shows favaourable physical properties for naphthalene as drug molecule. A drug molecule's physical and chemical properties decide its ability to traverse across tissue membranes. Main factors include molecular weight, molecular volume, and surface areas. The dipole moment of drug molecules decides the receptor interactions and its absorption. The present study evaluated these parameters of naphthalene using computational methods. Naphthalene showed satisfactory physical properties like globularity, surface area for accessibility of solvents, molecular volume, cohesive interaction index, and molecular dipole moment [Table 1 - see PDF]. The prediction of drug-likeliness of naphthalene was evaluated following ro5. Naphthalene showed very minimum number of violations against ro5 in terms of molecular weight, solubility, hydrogen bond donor, hydrogen bond acceptor, and rotatable bonds. The number of heavy atoms and rotatable bonds were minimum. Octanol/water coefficient decides the hydrophobicity, which is in satisfactory range for naphthalene. Hexadecane/gas partition coefficient also showed good polarizability by naphthalene [11-13]. There was no weak surface polarity in naphthalene (Table 2 - see PDF). The bioavailability descriptors of naphthalene satisfied Jorgensen's rule of three (ro3) [14-18]. This revealed the aqueous solubility, intestinal absorption and minimum metabolite formation of naphthalene, which implies its oral absorption and bioavailability (Table 3 - see PDF). The CNS activity of naphthalene was revealed by blood/brain partition coefficient descriptor, which is in recommended range [18]. The computed MDCK tissue permeability was also maximum which implies traversal of naphthalene across cell membranes (Table 4 - see PDF). Naphthalene showed dermal penetration rate of 11.5 µg cm-2 hr-1, which is poor as per standard reference value (Table 5 - see PDF). This is directly related to skin permeability (Kp), aqueous solubility (S), and molecular weight of the compound, and calculated as J_m_ = K_p_ X MW X S 

Plasma-protein binding decides the distribution of drug in the body [10]. The computed serum albumin protein binding of naphthalene was satisfactory, and in standard reference range (Table 6 - see PDF). Naphthalene showed zero metabolite formation, which implies that it reaches the target site unchanged (Table 7 - see PDF). In contrary, naphthalene-producing metabolites like 1,2-naphthquinone and 1,2-dihydroxynaphthalene were demonstrated in rabbit experiment. These metabolites were shown to have active interactions with many enzymes in the body causing toxic effects [19]. Few other studies revealed naphthalene metabolites from urine samples of four species fed with naphthalene. They found 1-naphthol, 2-naphthol, 1:2-dihydronaphthalene-1: 2-diol, 1:2-dihydro-2-hydroxy-1-naphthylglucosiduronic acid, 1-naphthylmercapturic acid in urine samples analyzed with chromatography techniques [20-22]. In-vitro study using bacterial cultures treated with naphthalene showed metabolites like 2-naphthoic acid, decahydro-2-naphthoic acid, 5,6,7,8-tetrahydro-2-naphthoic acid, octahydro-2-naphthoic acid through mass spectroscopy analysis [23]. The toxicity profile of this metabolite varied as revealed by an in-vitro study. The primary metabolites were less toxic to leucocytes compared to naphthalene-1, 2-epoxide, in causing glutathione depletion and genotoxicity [24]. Naphthalene has cardiac toxic potentiality revealed through the HERG K+ channel blockade descriptor [25].

## Conclusion

We document the computer aided pharmacokinetic profiling and toxicity analysis data of naphthalene.
